# Schmallenberg virus: a systematic international literature review (2011-2019) from an Irish perspective

**DOI:** 10.1186/s13620-019-0147-3

**Published:** 2019-10-09

**Authors:** Áine B. Collins, Michael L. Doherty, Damien J. Barrett, John F. Mee

**Affiliations:** 1 Animal and Bioscience Research Department, Teagasc, Moorepark, Fermoy, Co, Cork, Ireland; 2 School of Veterinary Medicine, University College Dublin, Dublin 4, Ireland; 3 Department of Agriculture, Surveillance, Animal By-Products and TSE Division, Food and the Marine, Backweston, Celbridge, Co. Kildare Ireland

**Keywords:** Schmallenberg virus, Ireland, Arbovirus, *Culicoides*, Domestic Ruminants, Review

## Abstract

In Autumn 2011, nonspecific clinical signs of pyrexia, diarrhoea, and drop in milk yield were observed in dairy cattle near the German town of Schmallenberg at the Dutch/German border. Targeted veterinary diagnostic investigations for classical endemic and emerging viruses could not identify a causal agent. Blood samples were collected from animals with clinical signs and subjected to metagenomic analysis; a novel orthobunyavirus was identified and named Schmallenberg virus (SBV). In late 2011/early 2012, an epidemic of abortions and congenital malformations in calves, lambs and goat kids, characterised by arthrogryposis and hydranencephaly were reported in continental Europe. Subsequently, SBV RNA was confirmed in both aborted and congenitally malformed foetuses and also in *Culicoides* species biting midges. It soon became evident that SBV was an arthropod-borne teratogenic virus affecting domestic ruminants. SBV rapidly achieved a pan-European distribution with most countries confirming SBV infection within a year or two of the initial emergence. The first Irish case of SBV was confirmed in the south of the country in late 2012 in a bovine foetus.

Since SBV was first identified in 2011, a considerable body of scientific research has been conducted internationally describing this novel emerging virus. The aim of this systematic review is to provide a comprehensive synopsis of the most up-to-date scientific literature regarding the origin of SBV and the spread of the Schmallenberg epidemic, in addition to describing the species affected, clinical signs, pathogenesis, transmission, risk factors, impact, diagnostics, surveillance methods and control measures. This review also highlights current knowledge gaps in the scientific literature regarding SBV, most notably the requirement for further research to determine if, and to what extent, SBV circulation occurred in Europe and internationally during 2017 and 2018. Moreover, recommendations are also made regarding future arbovirus surveillance in Europe, specifically the establishment of a European-wide sentinel herd surveillance program, which incorporates bovine serology and *Culicoides* entomology and virology studies, at national and international level to monitor for the emergence and re-emergence of arboviruses such as SBV, bluetongue virus and other novel *Culicoides*-borne arboviruses.

## Materials and methods

In order to ensure a systematic, up-to-date review of the literature the following search strategy was implemented. Harzing’s *Publish or Perish* software (Windows GUI Edition) 7.10.2373.7118 [1] was used to search and extract relevant literature from the online databases Google Scholar. The review keywords “Schmallenberg virus, *Culicoides*, ruminants Ireland” (and their combinations using AND/OR) were entered in the *Publish or Perish* software. All publications between the years 2011 (the year Schmallenberg virus was first identified) and 2019 were searched. No restrictions on language were imposed so long as an English abstract was available. All relevant publications were added to the master list (*n* = 576). Each publication was then critically evaluated (removing duplicates) to determine whether it had information which met the aim of this review or not; all relevant publications were then selected for possible inclusion in this review. The bibliographies within these publications were also searched for further relevant publications. In total, 226 publications met the inclusion criteria set out in the aim of the literature review and so were cited.

## Background

Emerging infectious diseases, particularly those caused by novel emerging pathogens, are causes for concern to human and animal health globally; approximately 75% of emerging infectious diseases are zoonotic, originating principally from wildlife [2]. Similar to the emergence of bluetongue virus (BTV) in Northern Europe (2006), the recent and unprecedented emergence of Schmallenberg virus (SBV) in Germany in 2011 has highlighted the susceptibility of domestic livestock and wildlife throughout Europe to arboviruses from distant geographical regions.

During the summer and autumn of 2011, a previously unknown disease was reported in adult dairy cattle in Germany and the Netherlands [3]. The disease was characterised by the non-specific clinical signs of pyrexia, drop in milk yield and sometimes diarrhoea; however, no known agent could be implicated in the affected cattle. Diagnostic tests excluded a wide range of classical endemic and emerging viruses, such as pestiviruses, bovine herpes virus type I, foot-and-mouth disease virus, bluetongue virus, epidemic haemorrhagic disease virus, Rift Valley fever virus, and bovine ephemeral fever virus, as the causative agent [3].

Genomic analyses conducted at the Friedrich-Loeffler-Institut, Germany, on a pool of blood samples collected from three acutely infected cows identified sequences of a novel virus in autumn 2011. This new virus was named Schmallenberg virus (SBV) after the town Schmallenberg (North Rhine-Westphalia) near which the affected animals originated [3]. Phylogenetic analysis demonstrated that SBV is a member of the Simbu serogroup in the Orthobunyavirus genus of the Peribunyaviridae family (order Bunyavirales). This was the first report to confirm the emergence of a Simbu serogroup virus in Europe [3]. Other members of this serogroup include Akabane and Aino viruses (*vide infra*). In December 2011 and January 2012, an epidemic of congenital malformations was identified in domestic ruminants in Germany [4, 5] and the Netherlands [6]. Diagnostic tests on brain tissue samples collected from malformed lambs and calves confirmed SBV infection. It soon emerged that SBV is a teratogenic virus; pregnant female ruminants (cattle, sheep, goats) which became infected with SBV during the summer and autumn of 2011 subsequently gave birth to congenitally infected and malformed offspring. In addition, virus detection studies confirmed SBV infection in a range of *Culicoides* arbovirus vectors, implicating *Culicoides* species in the transmission of the virus [7].

**Fig. 1:**
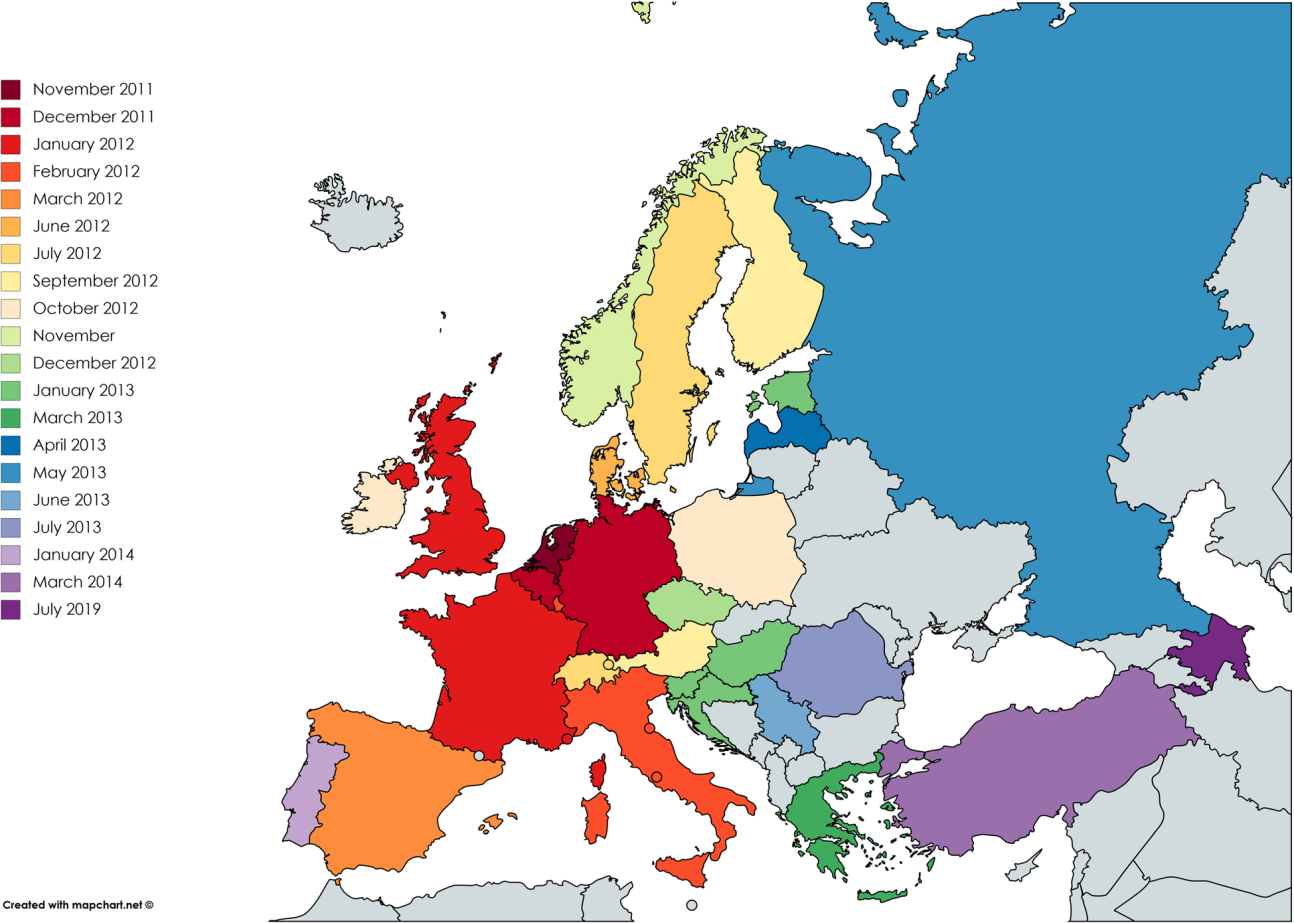
Schmallenberg disease distribution by country and date of initial report of detection by serology and/or RT-qPCR. Map created with mapchart.net ©

During the spring of 2012, a number of other European countries including France, the United Kingdom (UK), Luxemburg, Italy and Spain reported confirmed cases (clinical/pathological signs and PCR- positive) of SBV infection in malformed calves, lambs and goat kids. Later in 2012 and in 2013, cases of congenital Schmallenberg disease were confirmed in a number of countries across Europe [8–10] (Fig. 1).

The first Irish case was confirmed in late October 2012 in a bovine foetus at the Cork Regional Veterinary Laboratory [11]. Subsequently, congenitally malformed calves and lambs displaying lesions consistent with SBV were confirmed by PCR in the latter months of 2012 and up to May 2013 [11]. These cases were in the south and south east of the country [11]. A national Irish serosurvey conducted in late 2012 demonstrated that much of the south and south east of the country had been exposed during 2011 and 2012, while the north and north west remained predominantly unexposed [12]. A bulk-tank milk (BTM) surveillance study found no evidence of further spread of SBV in dairy herds in the south west of Ireland during 2013 [13]. This is in contrast with other European countries such as Germany [14] and Belgium [15], where SBV appeared to re-emerge in cattle herds and sheep flocks during 2012-2013, albeit at a considerably lower level compared to 2011-2012. In the four years (2012 to 2015) following the initial European Schmallenberg epidemic there were a number of reports of SBV overwintering and continued virus circulation at low levels in several European countries [16–19].

However, there was little or no evidence of SBV circulation in Ireland in the three years (2013-2015) following the initial emergence of SBV in Ireland in 2012 [20]; a similar situation was present in the UK [21] and in the Netherlands [22]. In Ireland it is possible that a high herd immunity (animal-level seroprevalence was 62.5 % in spring 2014) may have reduced the SBV’s ability to circulate in animals in previously exposed herds in the vector seasons of 2014 and 2015 [20]. SBV is typically highly efficient in spreading in herds in the presence of transmitting vector species [23, 24]. This is due to the rapid rate of virus replication and the high probability of transmission from host to vector [25]. This is supported by evidence of high within-herd seroprevalence in cattle and sheep after the 2011/2012 Schmallenberg epidemic [26]. These epidemiological characteristics of SBV result in a high basic reproductive number (R_0_), estimated to be as high as 6.2 for cattle-only farms and 7.6 for sheep-only farms [25], which reduces the probability of finding low numbers of seropositive animals once the virus has circulated in a naïve ruminant population [20]. Using this R_0_ value, it is interesting to note that the effective reproductive number (R_e_ = R_0_ × fraction of the population of susceptible animals) in animals in previously exposed herds in Ireland in spring 2014 (one year after the Irish Schmallenberg epidemic) would have been greater than one (Re = 2.33; Re = 6.2 × 0.375) highlighting the potential for SBV to re-circulate in these herds during 2014 and subsequent vector seasons, despite a perceived high herd immunity [20].

**Fig. 2:**
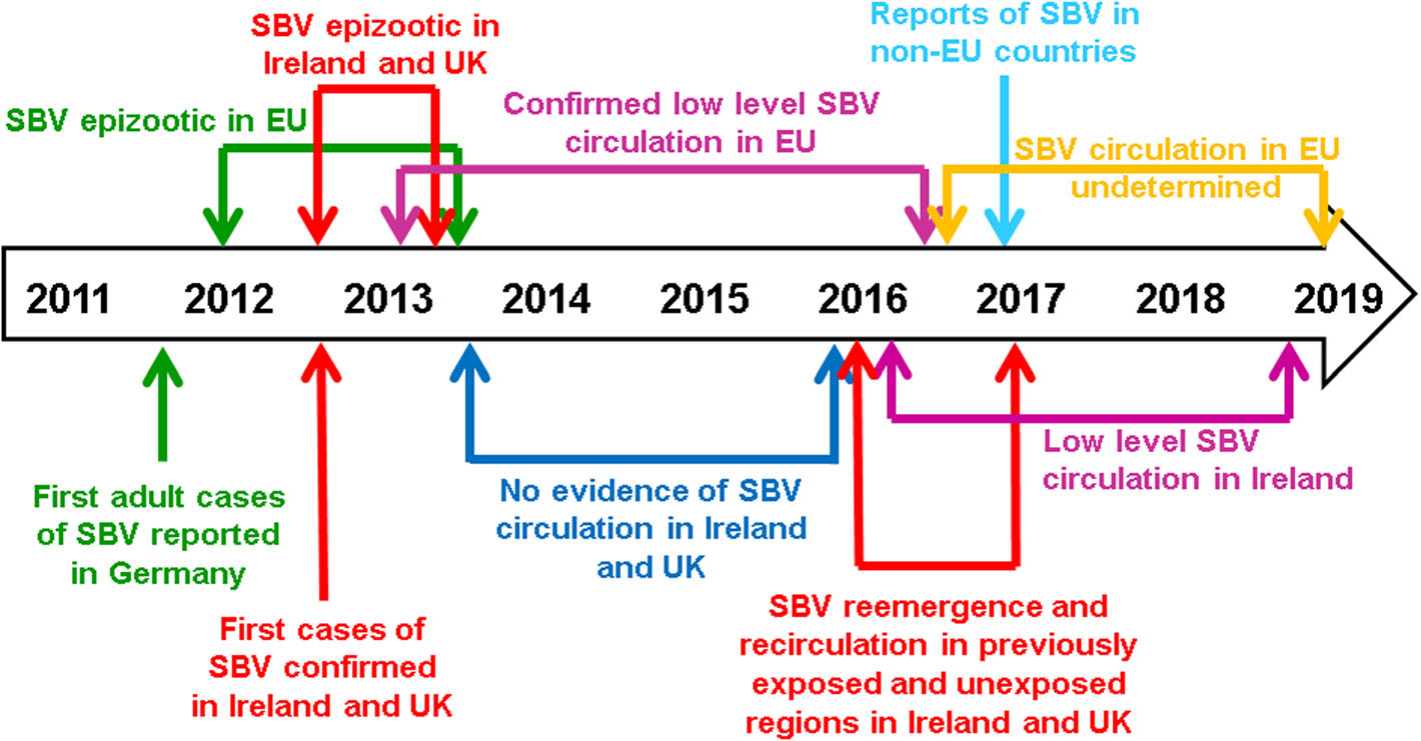
Schmallenberg virus timeline between 2011 and 2019

In 2016, SBV re-emerged and recirculated in Ireland, the UK and in Belgium [27–29] resulting in a second outbreak of abortions and congenital malformations in ruminants. SBV was also confirmed in regions that had previously been unaffected by SBV (north and north west of Ireland [30]), and in countries outside of the EU [31–38], highlighting the continued geographical expansion of SBV. The virus continued to circulate in Ireland and the UK in 2017, which is in contrast with other European countries which have not (yet, September 2019) reported notable SBV circulation during this time (Fig. 2) [39]. Further research is recommended to determine the extent of SBV circulation in continental Europe during this time.

### The pathogen

#### Virus structure and genome

Similar to other members of the Peribunyaviridae family, SBV is an enveloped virus with a genome composed of three negative-sense single-stranded RNA segments which are named according to their size: Small (S), Medium (M) and Large (L) [40]. It is assumed that these segments have a similar coding capacity and proteins to other related Orthobunyaviruses [41]. Considering that SBV is an arbovirus, antigenic drift in the virus genome is considered limited because the virus relies on vector transmission; mutations which could be advantageous for replication in the final host might be disadvantageous in the vector [42]. This bottleneck has also been seen with other RNA viruses that are transmitted by mosquito vectors, such as Venezuelan equine encephalitis virus [43]. This is supported by reports of high SBV genetic stability in vector-derived SBV sequences in Poland [44]. Additionally, Hoffmann et al. (2015) reported that SBV has a relatively low mutation rate both in vitro and in vivo [45]. Several field studies of virus variability have also demonstrated that the virus is relatively stable over time [46, 47]. Some variations in the SBV genome have been reported in the time since the virus first emerged in Europe in 2011 [48–50]. Of the three SBV RNA segments, the M-segment, which encodes two surface glycoproteins (glycoprotein n; Gn and glycoprotein c; Gc) and one non-structural protein (NSm), is considered the most variable segment within the SBV genome. Similar to Akabane virus [51], the M segment of SBV possesses a hypervariable region within the sequence coding for glycoprotein Gc, lending it to be the most variable segment within the genome [48, 49, 52]. The SBV L-segment encodes RNA-dependent RNA polymerase [53, 54] while the S-segment encodes the nucleoprotein N and the non-structural protein NSs [49].

#### Virus classification and origin

Schmallenberg virus (SBV) is a Simbu serogroup virus in the genus Orthobunyavirus (Family Peribunyaviridae, order Bunyavirales) [53]. There are over 170 viruses within the Orthobunyavirus genus including viruses responsible for disease in humans (Oropouche virus, La Crosse virus) and in ruminants (Akabane virus, Aino virus, Cache Valley Fever virus). The Simbu serogroup viruses are prevalent in Africa, Oceania, and the Middle East and include more than 25 viruses such as Oropouche, Simbu, Akabane, Douglas, Sathuperi, Aino, Shamonda and Peaton viruses [53]. Akabane virus (AKAV) [55, 56], Aino virus (AIV) [57, 58] and Shamonda virus (SHAV) [59, 60] can cause similar clinical signs and pathology to Schmallenberg virus (*vide infra*) [26].

When SBV was first discovered in 2011, metagenomic analyses revealed that the virus shared similar genomic sequences with three other Orthobunyaviruses which also infect cattle; Shamonda (SHAV), Aino (AIV) and Akabane (AKAV) viruses [3]. None of these viruses have been detected in Europe to date (September 2019). Interestingly, SBV sequences share a 69% identity with the Akabane virus L-RNA-segment, 71% identity with the Aino virus M-RNA-segment and 97% identity with the Shamonda virus S-RNA-segment [3]. Phylogenetic analyses of SBV have revealed that it may be a reassortant virus with the M RNA-segment from Sathuperi virus and the S and L RNA-segments from Shamonda virus [60]. However, one study based on phylogenetic and serologic analyses, produced conflicting results with Yanase et al. (2012) [60]; Goller et al. (2012) suggested that SBV belongs to the Sathuperi virus species and may be an ancestor of Shamonda virus [61]. Understanding the exact phylogeniology of SBV has proven challenging, particularly considering the fact that a number of the Simbu viruses have yet to be fully sequenced. Further research in this area is recommended; understanding the phylogeniology of SBV may help elucidate the origin and the epidemiological circumstances surrounding the emergence of this novel virus in Europe.

Currently (September 2019), the geographical origin of SBV also remains unknown. The distribution of closely related Simbu serogroup Orthobunyaviruses (Aino, Akabane, Sathuperi and Shamonda viruses) in Africa and Australasia [59] suggest that SBV may have been introduced into Europe from distant geographical regions. Some reports suggest that aircraft transporting animals from an area where SBV may be enzootic or in virus-infected midges may be involved in the introduction of SBV into Europe [62]. It is interesting to note that SBV emerged in a similar geographical location (triangular region located at the border of Germany, Belgium and The Netherlands) to where bluetongue virus serotype 8 (BTV-8) emerged 5 years previously [63]. One could hypothesise that both SBV and BTV-8 were introduced into Europe via a similar, but not yet defined, route [64, 65]. A model demonstrated that the majority of SBV infections in the UK in 2012 occurred as a result of SBV-infected midges being transported through downwind movement facilitated by prevailing winds from continental Europe [66], most likely from Northern France [67]. Similarly, the emergence of SBV in Ireland in 2012 has been attributed to the transport of SBV-infected *Culicoides* in wind movements from Southern England [68]. Interestingly, when SBV sequences collected during the SBV re-emergence in the UK in 2016-2017 were compared with those originally isolated in the UK in 2012, a second distinct clade of SBV was identified; further research is required to determine if these novel viruses represent a re-incursion from continental Europe or were derived through spatial separation of viruses already present in the UK [67].

### Host range

#### Ruminant species

Schmallenberg virus primarily infects ruminants. Direct and indirect detection of SBV in has been identified in cattle, sheep and goats (either in adult animals or in their offspring) [4–6, 69]. SBV RNA or antibodies have been detected in a wide range of wild and exotic ruminants (alpacas, elk, State Anatolian and Congo water buffalo, European bison, red deer, fallow deer, roe deer, sika deer, llama, reindeer, moufflon, water buffalo and chamois) and camelids in a number of European countries including France, Germany, the Netherlands, Sweden and the UK [26, 70, 71], and in wild (fallow, red and sika) deer in Ireland [72]. In Spain, SBV antibodies were also confirmed in wild (red, fallow) deer and also wild moufflon [73]. Experimental infection with SBV in alpacas and llamas resulted in sub-clinical infection with detectable SBV-RNA viraemia for 3 to 7 days post inoculation [74]. In Poland, a cross-sectional study of Schmallenberg virus seroprevalence in wild ruminants at the end of the vector season of 2013 suggested that wild ruminants might play a role in SBV transmission, however the lower seroprevalence in relation to the domestic ruminants suggests the spill-over effect from the latter, rather than inverse [75]. SBV antigen was also confirmed in the serum of a 6-month-old elk found in Białowieża national park in Poland, however no clinical sign were observed [76]. This may be due to the free-ranging nature of these wild animals and the limited contact with humans. In Belgium, Italy and France, SBV antibodies were also confirmed in wild ruminants (wild cervids and wild alpine ungulates) [77–80]. Additionally, serum samples collected from a wide range of zoo ruminants have also tested positive for SBV antibodies in the UK [26, 81], in France and in the Netherlands [82]. In contrast to domestic ruminants, clinical signs associated with SBV infection have not been described in these wild and exotic species; further investigation is required to clarify the effects of SBV infection in these species and to assess their role in the epidemiology of SBV [75, 76].

#### Non-ruminant species

Owing to the fact that a number of viruses in the Orthobunyavirus genus can cause disease in humans, one of the most critical questions regarding SBV during the initial epidemics was whether SBV transmission from animals to humans was possible. Molecular and serological investigations in highly exposed human populations in Germany and in the Netherlands revealed no evidence of SBV infection; neither SBV-RNA nor antibodies were detected [83, 84]. Evidence of indirect SBV infection has been reported in a range of non-ruminant species. SBV-specific antibodies have been detected in free-ranging wild boar in a number of countries including Germany [85], Belgium [86], Poland [87] and Spain [73]. A limited number of reports suggest that SBV infection can occur in dogs. In France SBV-RNA was detected in the brain of a puppy with torticollis and degenerative encephalopathy, while SBV antibodies were detected in its mother [88]. SBV antibodies were also reported in one dog without clinical signs in Sweden [89]. However, SBV serological investigations in 132 dogs in Belgium [90] and in wild carnivores in Germany [85] found no evidence of SBV infection. Experimental SBV infection in pigs leads to transient seroconversion, however no SBV-RNA was detected [91] suggesting they may become infected but do not develop disease. SBV antibodies have also been detected in exotic zoo species (onager, Grevy’s zebra, Asian elephant and babirusa) in two zoological parks in the UK [26, 81]. Previously, The European Food Safety Authority considered that horses did not play a role as a reservoir in the epidemiology of SBV [26]. However, one recent study reported the detection of SBV-specific antibodies in horses in Iran; this is the first reported evidence of SBV infection in equine species [35]. However, the results of this study should be interpreted with caution; these positive results were not confirmed using virus neutralisation tests and SBV RNA was not detected [35]. Moreover, the animals tested originated in the Simbu serogroup endemic areas of northern and northeast Iran; the possibility of cross-reaction with other related Simbu serogroup viruses such as Shuni (SHUV), Aino (AINV), and Akabane (AKAV) virus could not be excluded [35].

### Clinical signs and lesions

In female dairy cattle (pregnant and non-pregnant), SBV infection can cause short-duration, non-specific and somewhat inconspicuous clinical signs such as pyrexia (fever up to 41°C), drop in milk yield and diarrhoea [3]. Clinical signs in adult cattle typically last for up to 6 days and are closely associated with the short-duration SBV viraemia [3, 92]. In small ruminants SBV infection is typically subclinical, however clinical signs such as fever, decreased milk production and diarrhoea are reported to occur during the viraemic phase [93]. Congenital foetal Schmallenberg virus infection in naïve ewes and goats can result in abortions and stillbirths with or without congenital malformations [93]. In goats, one study reported a reduction in milk yield of up to 50% in the lactating flock [94]. In sheep flocks anecdotal evidence suggests that lactating sheep also experienced fever, diarrhoea and decreased milk production, however a conclusive link to SBV infection could not be determined [95]. In sheep experimentally infected sheep with SBV, one RNAemic sheep showed diarrhoea for several days, but fever was not recorded in any of the animals [92]. Experimental infection with SBV in adult female (non-pregnant) goats and bucks resulted in seroconversion, but inoculated goats did not display any clinical signs, gross lesions or histological lesions, nor was SBV RNA detected semen samples collected from two virus inoculated bucks [96].

**Fig. 3:**
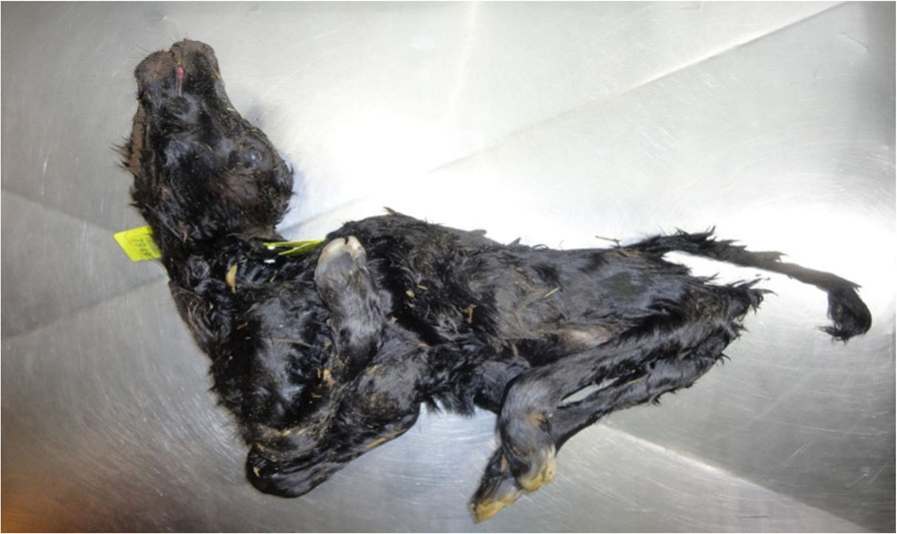
Bovine foetus congenitally infected with Schmallenberg virus presenting with severe arthrogryposis of all four limbs, torticollis, kyphosis, brachygnathia inferior, and skeletal muscle hypoplasia. Image courtesy of Dr. John Mee, Teagasc, Ireland

**Fig. 4:**
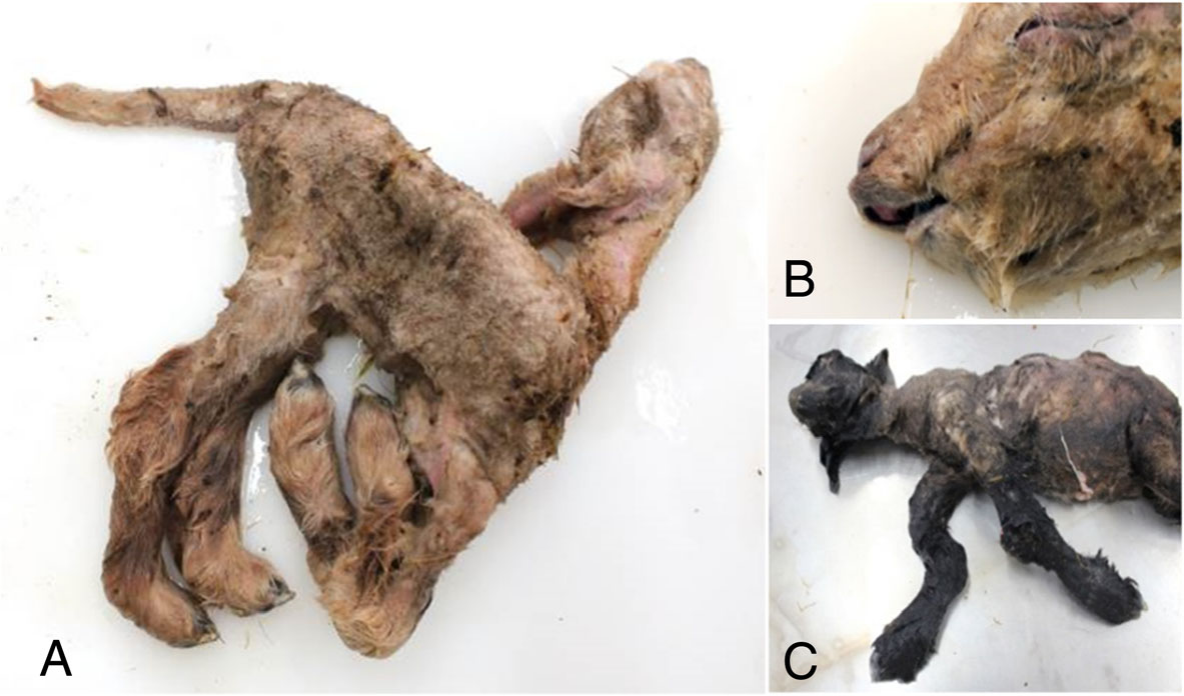
Ovine foetuses congenitally infected with Schmallenberg virus; image (**a**) & (**b**) from the same lamb presenting with severe arthrogryposis of all four limbs, torticollis, brachygnathia infectior and skeletal muscle hypoplasia. Image (**c**) of a second lamb presenting with scoliosis and bending and twisting of the forelimbs. Images courtesy of Cosme Sanchez Miguel, DAFM, Ireland

The most notable clinical signs associated with SBV infection are those observed in the offspring of animals that become infected during the critical stage of gestation resulting in congenital Schmallenberg disease. As described for AKAV and AIV, *in-utero* infection with SBV can cause abortions, premature or stillbirths, mummified foetuses and congenitally malformed offspring characterised by the Arthrogryposis-Hydranencephaly-Syndrome (AHS) (Figs. 3 and 4). Subsequent to the emergence of SBV in Germany in 2011, SBV was implicated in an epidemic of congenitally malformed lambs in the Netherlands; SBV antigen was detected in approximately 50% of brain tissue samples collected from affected lambs [6]. This was the first report to demonstrate the ability of SBV to cross the placenta in and induce teratogenic effects in developing embryos/foetuses [6].

SBV infection during early gestation in cattle has also been associated with embryonic mortality and return to oestrus [97–99]. This is supported by reports of reduced fertility in SBV-affected herds in Switzerland (increased number of inseminations per cow) [100], in Germany and in the Netherlands in 2011 (repeat oestrus, increased number of abortions and short gestations and an increase in the number of artificial inseminations per cow) [99]. In ruminants, the primary pregnancy recognition signal is an interferon (IFN-tau); interestingly a number of studies have shown that the NSs protein of SBV plays a major role in inhibiting IFN production and contributes to SBV pathogenesis [101–103]. Reports of abortions and increased numbers of repeated oestruses suggestive of early embryonic mortality have also been attributed to SBV infection in sheep flocks [95, 97, 104, 105] and goat flocks [94, 97]. In Ireland when SBV re-emerged during 2017, one dairy farmer reported severely reduced fertility; it was estimated that 25% of herd, which had previously been confirmed pregnant, had aborted [106].

In pluriparous animals, the birth of one congenitally infected offspring and one non-infected offspring has been reported in SBV-infected calves [69] and lambs [6]. Congenitally infected SBV neonates can also survive birth; congenitally infected lambs have been reported to present with a range of clinical signs such as malformations, “dummy lambs” unable to suckle, weak lamb syndrome, non-specific neurological signs and normal presentation [6, 107]. One report of a calf born at full term describes a range of neurological signs also; hypertonicity, hyperreflexis, depression, blindness, ventrolateral strabismus and inability to stand [108]. These neurological signs, in addition to reports of musculoskeletal malformations, indicate that SBV has a tropism for the central nervous system (CNS) and musculoskeletal system in developing embryos. Target cells of SBV infection include neurons in the cerebral cortex, brainstem and spinal cord [102, 109]. Varela et al. (2013) report that malformations and deformities observed in SBV-infected lambs and calves are accompanied by muscle hypoplasia and demyelination; SBV appeared to infect the neurons of the grey matter of the spinal cord, suggesting that muscular hypoplasia and muscular defects observed in SBV infected lambs and calves are mostly secondary to damage of the central nervous system (CNS) [102].

Unlike AKAV, where there are a small number of reports of a highly virulent AKAV strain known as the Iriki strain which can cause encephalomyelitis in naturally infected adult cows [110], there are no reports to date of SBV-associated lesions in naturally infected non-pregnant adult cattle other than pyrexia, diarrhoea and milk drop. However, a range of pathological findings are reported in congenitally infected foetuses and neonates with presumptive or confirmed SBV infection (Table 1). The severity of congenital Schmallenberg virus CNS lesions may be greater in lambs than in calves [109], this may be due to the shorter length of gestation in sheep (approximately 5 months) compared to cattle (approximately 9 months).

**Table 1:** Reported pathological findings associated with natural congenital Schmallenberg virus infection in domestic ruminants (bovine, ovine and caprine)

Species	Gross pathology	Histopathology	References
Bovine	Head and CNS	Non-suppurative meningo-encephalitis,	[108, 109, 111, 112]
	Porencephaly, hydranencephaly, brain stem hypoplasia, cerebellar hypoplasia, cerebellar dysplasia, micromyelia		
		Non-suppurative poliomyelitis, skeletal muscle hypoplasia, lymphoid depletion in thymus and lymph node, chronic hepatitis	
	Skeletal		
	Arthrogryposis, torticollis, lordosis, scoliosis, kyphosis, cranial malformations, brachygnathism inferior, prognathia		
	Visceral		
	Ectopia cordis, pulmonary hypoplasia, ventricular septal defect		
Ovine	Head and CNS	Non-suppurative meningo-encephalitis, skeletal muscle hypoplasia, lymphoid depletion in spleen or lymph node, cataract, haematopoietic cellularity in bone marrow	[4, 6, 109]
	Brachynathism inferior, domed skull, flattened skull, hydranencephaly, hydrocephalus, micrenencephaly, macrocephaly, brainstem hypoplasia, cerebral hypoplasia, cerebellar hypoplasia, cerebellar dysplasia, micromyelia		
	Skeletal		
	Arthrogryposis, torticollis, lordosis, scoliosis, kyphosis,		
	Visceral		
	Cardiac ventricular septal defect, unilateral hydronephrosis, colonic atresia		
Caprine	Head and CNS	Non-suppurative meningo-encephalitis, nonsuppurative poliomyelitis	[4, 113]
	Hydrocephalus, porencephaly, cerebellar hypoplasia		
	Skeletal		
	Arthrogryposis, vertebral deformities, brachynathism inferior		
	Visceral Pulmonary hypoplasia,		

This table is adapted from Doceul et al. (2017) [114]

The CNS, the axial skeleton and skeletal muscle are the most commonly reported sites for congenital malformations, individually or in combination, in domestic ruminants [4, 6, 109, 111]. Arthrogryposis can be pathognomonic for SBV infection and can be associated with skeletal muscle hypoplasia, with histological evidence of decreased number and diameter of myofibrils, with and without loss of cross-striation in myofibrils and fatty replacement [4, 111]. In the CNS, the most commonly reported lesions described are hydranencephaly, porencephaly, hydrocephalus, cerebellar hypoplasia and micromyelia (Table 1), with histological evidence of non-suppurative inflammation, neuronal degeneration and necrosis (Table 1). Malformations of the vertebral column can also be pathognomonic for SBV infection and include lordosis, kyphosis, scoliosis, kypho-scoliosis and torticollis (Table 1), a number of animals are also reported to have brachygnathia inferior [109].

### Pathogenesis

As SBV is a relatively newly discovered virus, the pathogenesis of Schmallenberg disease is poorly defined. This is further complicated by the fact that only a limited number of studies have been reported investigating the pathogenesis of SBV in domestic animals (Table 1).

**Fig. 5:**
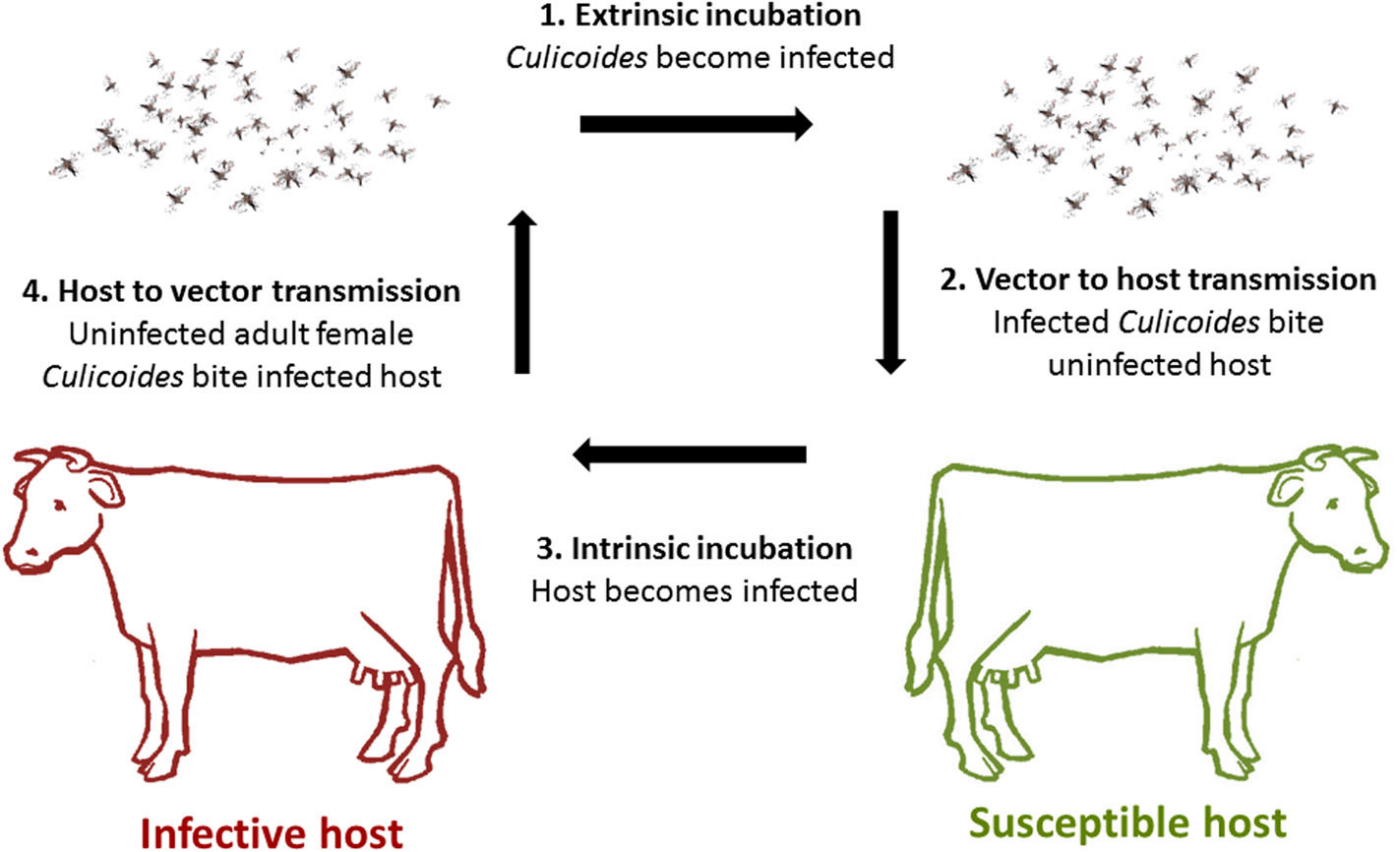
Arbovirus vector-mediated transmission cycle. (1) Extrinsic incubation period (EIP); the time during which an infected insect becomes infectious. (2) Vector-to-host transmission of virus through the bite of an infected *Culicoides*. (3) Intrinsic incubation period (IIP), the time during which the host becomes infectious. (4) Host-to-vector transmission of virus; when uninfected female *Culicoides* bite infected infectious hosts and become infected

When an adult animal is bitten by an infectious *Culicoides* vector, they can become infected with SBV and develop viraemia (Fig. 5), [3, 115]. SBV infection induces long-duration protective immunity in cattle [116, 117] and sheep [118]. However, if a naïve adult animal develops SBV viraemia during the critical stage of gestation (Fig. 5), the virus can cross the placenta and infect the foetus, possibly resulting in congenital Schmallenberg disease. While the gestation-susceptible period for AKAV has been defined in domestic ruminants (see Section Vertical Transmission *vide infra*), it has yet to be determined for SBV; it is assumed to be similar to that of AKAV [26]. However, a number of studies which have tried to elucidate the most susceptible stages of gestation for congenital SBV disease (based on the critical period for AKAV), have only led to foetal malformations in a very limited number of cases (Table 1).

The susceptibility of developing embryos/foetuses to SBV infection may depend on the maturity of the placentomes. Experimental infection with SBV in pregnant ewes at 45 days and 60 days of gestation resulted in placental colonization [119]; interestingly, in this study significantly more positive samples, from both extra-embryonic structures and lamb organs, were found in new-born lambs originating from ewes that were infected at day 60 compared to those infected at day 45 suggesting that that placentomes at 45 days of gestation are not sufficiently developed to sustain intensive viral replication, in contrast, placentomes at 60 days of gestation were [119]. Experimental infection with SBV in pregnant goats demonstrated vertical SBV infection during early pregnancy spanning at least the period between day 28 and 42 of gestation; this resulted in foetal mortalities, viable foetuses displaying lesions of the central nervous system, as well as viable foetuses without any detectable lesion [120]. There is no documented evidence of pathology in the developing embryo after AKAV infection in pregnant females during early gestation (first three weeks in small ruminants and the first two months in cattle), suggesting that the embryo may be protected from viral infection [56, 121]. The stage of development of the foetus and foetal immune system may also influence the outcome of foetal SBV infection. For example, studies have suggested that ovine foetuses may be more susceptible to SBV infection before the blood-brain barrier is developed (in sheep the blood-brain barrier starts to develop between days 50 and 60 of gestation and reaches full development by day 123) [102]. In AKAV-infected lambs, transplacental infection as early as 64 days of gestation can induce an immune response and the production AKAV-protective antibodies [122]. Similarly, pre-colostral SBV-specific antibodies can be detected in neonatal calves where the dam was (presumably) infected with SBV between day 47 and 162 of gestation [69].

The use of laboratory-based small animal models, principally mouse models, has facilitated research studies on the biology and pathogenesis of SBV. *In vitro* and *in vivo* studies have demonstrated that the SBV non-structural protein NSs is an important virulence factor [102] and has an important role in SBV pathogenesis [103, 123]. A mouse model has been developed in NIH Swiss mice to study SBV infection in brain tissue; intracerebral SBV inoculation resulted in deaths and severe brain lesions (malacia and haemorrhage of the cerebral cortex, multifocal vacuolation in the white matter of the cerebrum, and lymphocytic perivascular cuffing in the grey matter) [102]. Embryonated Chicken Egg models (ECEs) have been used previously to study the pathogenesis of a number of Simbu viruses [124–128]. More recently, Collins et al. (2018) demonstrated that ECE models are also suitable *in vivo* small animal models to study SBV infection [129]. Barry et al. (2014) demonstrated an association between activated caspase-3 (indicator of apoptosis) and SBV in the brain tissue of NIH Swiss mice inoculated intracerebrally [103]. Type I interferon receptor knock-out (IFNAR-/-) mice are also susceptible to SBV infection and can develop fatal disease and are thus a useful model to study SBV [130].

The effects of AKAV infection on developing embryos vary depending on the stage of gestation at which the dam becomes infected [131]. While, it is assumed this is similar for congenital SBV infection, further research is required to help elucidate the age-associated effects of SBV infection on developing embryos. The gestation-susceptible period for congenital AKAV infection is between day 28 and 56 days in small ruminants [55, 121, 122, 132], and between day 80 and 150 in cattle [56, 133–135]. However, experimental SBV infection in domestic ruminants during these dates has led to congenital malformations only in a very small number of cases (Table 2). This highlights the need for further research in this area; the development of suitable ruminant models to study SBV infection in developing embryos would be useful to help elucidate SBV pathogenesis.

**Table 2:** Overview of experimental and field studies with Schmallenberg virus in domestic ruminants contributing to our understanding of the efficacy of transplacental crossing, the capacity to induce congenital malformations, and the relationship between clinical and pathological malformations observed at birth in offspring and the moment of gestation at which the dam became infected

Study type		Virus strain	Species	No. studied	Inoculation route	Stage of gestation (day) at infection	Stage progeny examined	Major Outcome	Reference
Exp.	Field								
+		FLI inoculum^a^	Cattle	24	Sub-cutaneous	60-150	6 weeks PI	Preliminary data; indications for efficient placental crossing but limited capacity to induce malformations	Schmallenberg virus: Final Report EU, 2014^c^
+		FLI inoculum^a^	Cattle	11	Sub-cutaneous	105-120	10-28 DPI	Preliminary data; indications for efficient placental crossing but limited capacity to induce malformations	Schmallenberg virus: Final Report EU, 2014^c^
	+	Field	Cattle	71	Vector-mediated	13-162	At birth	Evidence of transplacental SBV infection only found in 13% of calves at birth; only 1 calf with malformations	[69]
+		Field	Cattle	36	Sub-cutaneous	60-150	10-35 DPI	SBV genome was detectable in at least one organ system of 18 out of 35 foetuses, but limited capacity to induce malformations	[225]
+		FLI inoculum^a^	Sheep	21	Sub-cutaneous	38-45	7 DPI	Preliminary data; indications of transplacental crossing in 64% of foetuses at 7 DPI; no malformations observed	Schmallenberg virus: Final Report EU, 2014^c^
+		FLI inoculum^a^	Sheep	28	Sub-cutaneous	45-60	At birth	Evidence of transplacental SBV infection only found in 14% of lambs at birth; no congenital malformations observed	[119]
+		FLI inoculum^a^	Sheep	17	Sub-cutaneous	45-60	At birth	Evidence of transplacental SBV infection; no congenital malformations observed; Placenta of 5 ewes contained infectious SBV at birth	[168]
+		FLI inoculum^a^	Goats	10	Sub-cutaneous	28-42	14-25 DPI	Preliminary data; several haemorrhagic and small foetuses observed after SBV infection	Schmallenberg virus: Final Report EU, 2014^c^
	+	Field	C/S/G	13	Vector-mediated	32-81	NS	13 cows with early foetal death after SBV infection	[98]

Table adapted from De Regge et al. (2017) [226]. *Exp* Experiment, *NS* Not specified, *C* Cattle, *S* Sheep, *G* Goats, *DPI* Days post inoculation

^a^Inoculum consisting of bovine serum collected 3 days post SBV infection, prepared and distributed by Friedrich Loeffler Institute

^b^Schmallenberg virus, March 2014, Technical and scientific studies, Final Report for the European Union Commission implementing Decision of 27 June 2012: available online at library.wur.nl/WebQuery/wurpubs/fulltext/310772

### Transmission

#### Arthropod vectors

SBV is an arthropod-borne virus transmitted by haematophagous arthropods from the *Culicoides* genus (Diptera: Ceratopogonidae). The arbovirus transmission cycle between vector and host is illustrated in Fig. 5. Uninfected *Culicoides* take a blood meal from a SBV-infectious host (ex: bovine, ovine, caprine). *Culicoides* become infectious (SBV replicates to transmissible levels within the midge) during the extrinsic incubation period (EIP), which is assumed to range between 9 and 41 days depending on microclimatic temperatures on farms [136]. Virus transmission from vector to host occurs when infectious female *Culicoides* bite immunologically naïve animals and transmit the virus via their saliva. The intrinsic incubation period for SBV can range from 2-6 days [3].

In order to implicate potential *Culicoides* species in SBV transmission, *Culicoides* specimens (entire specimens or heads only) can be tested for SBV RNA using RT-qPCR. Regarding entire specimen analysis, pigmented (parous; *Culicoides* that have oviposited and blood-fed at least once) *Culicoides* specimens are considered more appropriate for virus detection studies compared to nulliparous, blood-fed or gravid *Culicoides* specimens for a number of reasons. Pigmented (parous) *Culicoides* are likely to survive long enough for virus replication and dissemination within the vector and complete a full lifecycle (Fig. 5) resulting in full dissemination of the virus within the insect. In contrast, nulliparous *Culicoides* are not considered appropriate because they have not yet taken a blood meal from a mammalian host. However, it must be noted that nulliparous *Culicoides* can test positive for SBV RNA; this is suggestive of transovarial transmission within the vector [137, 138]. Blood-fed *Culicoides* are also considered inappropriate because positive detection of virus RNA could be a result from the blood meal in their mid-gut rather than fully disseminated virus infection in the midge (i.e. the virus may be present in the mid-gut but not present in the *Culicoides* salivary glands). While gravid *Culicoides* could be used in theory (as there is no longer a blood meal in the abdomen), positive detection of virus RNA could be ambiguous as it could be a result of residual virus present in their mid-gut from a previous blood meal, rather than a result of fully disseminated virus infection in the midge. An alternative method to analysing entire *Culicoides* specimens is to remove the head of the insect and analyse them separate to the body. While this method may be somewhat laborious, it would remove a certain degree of ambiguity regarding positive results in the case of entire *Culicoides* specimen analysis.

**Fig. 6:**
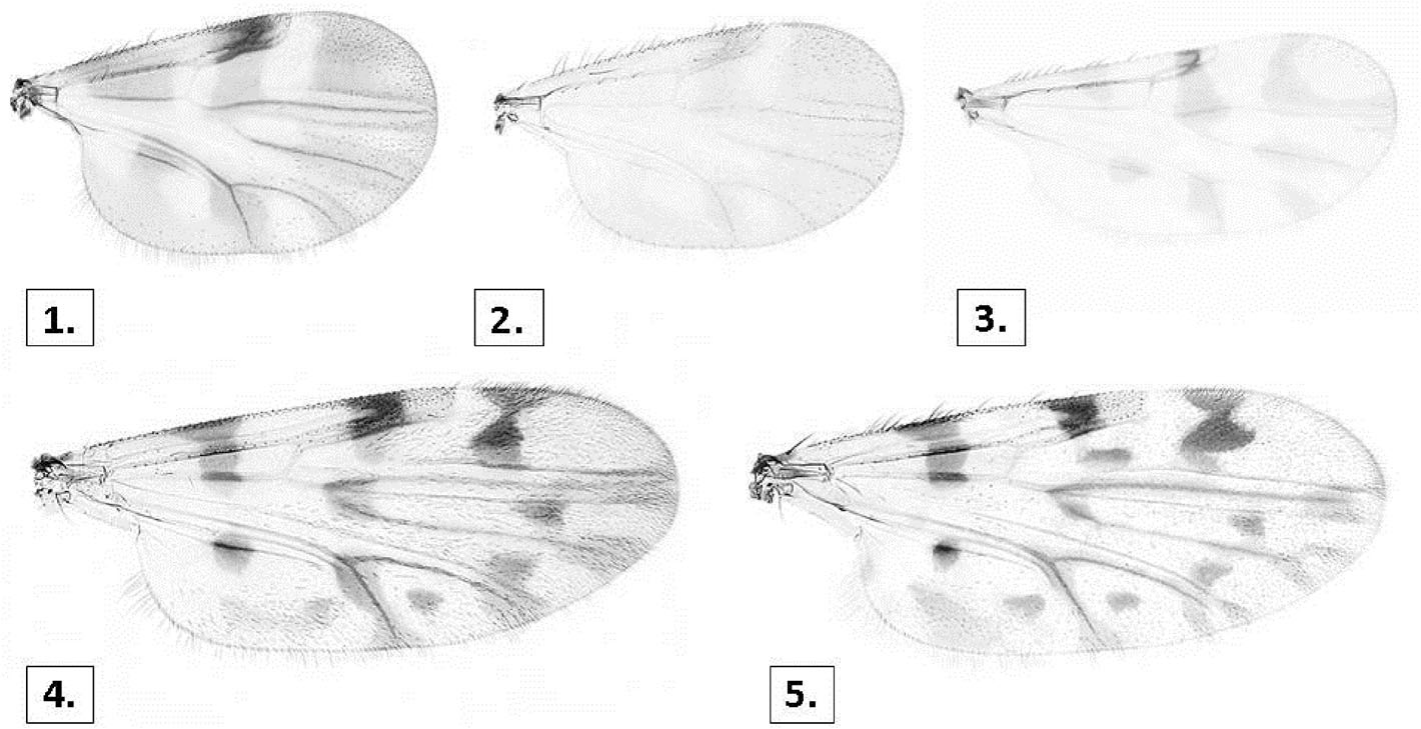
Wing patterns of *Culicoides* arbovirus vector species present in Ireland and the UK; (1) *C. obsoletus/C. scoticus*, (2) *C. chiopterus*, (3) *C. dewulfi*, (4) *C. pulicaris*, (5) *C. punctatus*. Images courtesy of The Pirbright Institute

Virus detection studies in field-caught *Culicoides* populations have implicated a range of Palearartic *Culicoides* in the transmission of SBV; SBV RNA has been detected in members of the *Culicoides* Obsoletus group (*C. obsoletus, C. scoticus, C. dewulfi* and *C. chiopterus*) and in members of the *Culicoides* Pulicaris group (*C. pulicaris* and *C. punctatus*), (Fig. 6) [7, 139–144]. SBV-RNA has also been detected in field-caught *C. nubeculosis*, *C. imicola, C. newsteadi, C. lupicaris* [144] (Note that *C. lupicaris* is currently considered a synonym of *C. delta* (preferred) by the UK reference laboratory), suggesting that these species may be involved in SBV transmission; however conclusive evidence of the potential role of these species in SBV transmission is required. Vector competence (the intrinsic ability of an arthropod to become infected, to support the development or replication of a pathogen, and to transmit it to a vertebrate host) has been demonstrated for both *C. obsoletus* and *C. scoticus* in laboratory experiments [145–147]. More recently, Pagès et al. (2018) demonstrated that *Culicoides* in the Obsoletus group and *C. imicola* are highly susceptibility to SBV infection in laboratory studies highlighting their role as competent SBV vectors [148]. Field data have confirmed the vector competence of *C. obsoletus, C. scoticus, C. dewulfi* and *C. chiopterus* [7, 149]. The role of mosquitos in SBV transmission has also been investigated and results indicate that they are not competent vectors; no evidence of SBV RNA was detected in 50,000 mosquitos trapped in Germany in 2011 [150], nor was there evidence for the persistence of Schmallenberg virus in overwintering mosquitoes in the Netherlands [151]. Experimental oral infection of two mosquito arbovirus vector species (*Aedes albopictus* and *Culex pipiens*) did not result in SBV replication to transmissible levels, indicating that these two species are not SBV arbovirus vectors [146]. In Germany, black flies (Diptera: Simuliidae) were also analysed for the presence of SBV and proved negative [152].

One study investigating *Culicoides* species in nine European countries demonstrated that the *Culicoides* Obsoletus ensemble (in this study the “*Culicoides* Obsoletus ensemble” refers to the *Culicoides* Obsoletus group and *C. dewulfi* together and includes the following species: *C. obsoletus*, *C. scoticus*, *Culicoides montanus* Shakirzjanova, *Culicoides chiopterus* (Meigen) and *C. dewulfi*) is widely distributed in Europe accounting for 83% of all specimens (8,842,998 specimens) identified [153]. In Ireland, Collins et al. (2018) demonstrated an abundance of putative *Culicoides* arbovirus vector species on Irish cattle farms, demonstrating the potential for future transmission of arboviruses among livestock on farms [154]. A Department of Agriculture, Food and the Marine (DAFM) *Culicoides* survey conducted in Ireland between 2007 and 2009 as part of the National BTV vector surveillance programme also indicated the presence of several *Culicoides* arbovirus vector species in Ireland [155].

A number of countries across Europe have established national surveillance systems to monitor *Culicoides* species and abundance to monitor for the (re-)emergence and (re-)circulation of arboviruses such as BTV. However, arbovirus surveillance programs which solely monitor vectors (*Culicoides* species) for evidence of infection are not considered the most effective programs to detect arbovirus circulation or emergence [20]. Virus detection (sensitivity) in insect specimens is frequently lower when compared to mammalian samples [20, 26]. Moreover, the detection of SBV in midges can be ambiguous as it does not always mean the virus is present at transmissible levels; bimodal distributions of cycle threshold (Ct) values for SBV in *Culicoides* sp. is reported suggesting that the virus can be present in the vector at transmissible and sub-transmissible levels [137, 149].

#### Vertical Transmission

In ruminants, if a pregnant female becomes infected with SBV (through a bite from infected *Culicoides*) she can develop a viraemia which may result in vertical transmission of SBV across the placenta into the foetus(es). Foetal infection with SBV during the critical stage of pregnancy can result in congenital Schmallenberg disease; the gestation-susceptible period for congenital Schmallenberg disease is not yet defined, it is assumed to be similar to that of Akabane virus; between day 28 and 56 days in small ruminants [55, 121, 122, 132] and between day 80 and 150 in cattle [56, 133]. Vertical transmission in cattle, sheep and goats has been demonstrated by the detection of SBV RNA in congenitally malformed neonates, stillborn and aborted foetuses [4, 6, 108, 112]. While SBV infection has been detected in non-ruminant species, vertical transmission has not been reported.

#### Horizontal transmission

Experimental subcutaneous inoculation of cows with SBV resulted in detectable concentrations of SBV RNA in faecal, oral and nasal swabs [92]. In contrast, experimental oral inoculation of cattle and nasal inoculation of sheep did not result in SBV RNA viraemia and animals remained seronegative [92, 115, 156, 157]. These findings suggest that direct transmission of SBV from one infected ruminant to another by direct contact, by oral route or by nasal route is unlikely. The possibility of venereal SBV transmission has not yet been fully determined; SBV has been detected in bovine semen samples collected from bulls naturally infected with SBV [158–162] however, it remains unknown whether females can become infected via this route. Experimentally infected goat bucks did not show evidence of SBV shedding in semen [96]. Interestingly, viraemia was produced in cows inoculated with Akabane virus into the uterus at oestrus [163], suggesting AKAV infected semen resulted in viraemia. Further research studies in this area using SBV are recommended.

#### Overwintering

SBV appears to have the ability to overwinter, that is, to survive for prolonged periods in vectors and/or in hosts during lower vector activity when no new hosts appear to become infected [26]. The mechanism by which this occurs is not fully understood. One of the first reports of SBV overwintering occurred during the winter of 2011-2012 in France; in May 2012, eight months following the most likely introduction date of SBV into France [164], evidence of acute infection was detected in cows suggesting SBV overwintered or was re-introduced during the winter of 2011-2012 [165]. Furthermore, in the three years (2012, 2013 and 2014) following the European Schmallenberg epidemic there were additional reports of SBV overwintering and continued virus circulation in several European countries [16, 17, 19, 140, 166].

A number of hypotheses have been proposed in order to help elucidate SBV overwintering. Overwintering in animal hosts has been explored though not yet proven, however this is unlikely due to the short-duration viraemia associated with SBV infection [3], and the low SBV RNA detection rates in malformed lambs and calves [167]. A recent study demonstrated that SBV can persist until birth in the placenta (cotyledons and intercotyledonary membranes) of ewes infected with SBV-infectious serum at day 45 and 60 of gestation [168]. This persistence of SBV for at least 100 days in pregnant ewes is a new aspect of SBV pathogenesis and could help to explain how SBV overwinters the cold season in temperate climate zones [168]. There is no documented evidence of persistently infected foetuses in pregnant hosts as seen with Bovine Viral Diarrhoea virus (BVDv) persistently infected animals.

Limited data suggest that SBV may overwinter in *Culicoides* vectors, possibly via transovarial transmission; two studies report the detection of SBV RNA in nulliparous *Culicoides* (*C. obsoletus/C. scoticus*, *C. pulicaris* and *C. punctatus*) [137, 138] and also in a pooled sample of male *C. scoticus* specimens [138]. These findings suggest the potential for transovarial transmission of SBV in arbovirus vectors; this may play an important epidemiological role in SBV overwintering. Overwintering in adult midges during the winter has also been considered. Adult midges of the *Culicoides* Obsoletus group are able to survive for long periods (for up to 10 days at 4°C and up to 92 days at temperatures ranging between 17°C and 35°C) without a blood meal [169]. These findings suggest that infected midges could persist during cooler months of the year and infect hosts once temperatures rise to temperatures that are more favourable for virus transmission. This is supported by evidence of SBV transmission in Germany in the winter of 2013 following a brief rise in temperature above 5°C for a couple days [23].

### Risk factors

The risk of SBV infection in domestic ruminants appears to differ among farm animal species, for example: within-group seroprevalence was typically lower in sheep flocks compared to cattle herds in Germany [69, 170, 171] and SBV seroprevalence was lower in goats compared to sheep in Belgium [172]. One study demonstrated that goats have a lower risk of SBV infection compared to sheep [171]. These findings suggest that the risk of SBV infection may be due to the inherent differences in the susceptibility of domestic ruminants to SBV infection. An alternative explanation may be due to *Culicoides* host species preferences. Cattle are the preferred host for the most common *Culicoides* arbovirus vector species [173, 174]. The differences in the exposure of host species to *Culicoides* arbovirus vectors, for example, different housing conditions and farming practices should also be considered. Dairy cattle herds managed outside appeared to have higher SBV seroprevalence compared to herds kept indoors, most likely due to the lower midge activity indoors [99, 175]. Similar results were also found in goats where flocks managed outdoors had high within-herd SBV seroprevalence [94, 176].

Timing of the reproductive season, particularly in sheep flocks with early breeding seasons (which overlapped with the *Culicoides* vector-active season), was also identified as a risk factor for congenital Schmallenberg disease [95]. As SBV infection induces long-duration immunity in cattle [116, 117] and sheep [118] (assumed life-long based on other Simbu Orthobunyaviruses), the age of the animal is also considered a risk factor for SBV infection; animals born after the initial Schmallenberg epidemic (after 2013) and animals which were not exposed to SBV in the years following the initial epidemic (2013-2019), are considered most at risk of infection [20, 22].

### Impact

The primary clinical impacts of SBV infection in domestic ruminants (cattle, sheep and goats) are embryonic mortality, abortions and congenital malformations of foetuses which become infected with the virus *in utero* [26, 97, 177, 178]. The impact of SBV infection on adult animals is primarily due to dystocia in cattle and sheep as a result of malformed calves and lambs [178] and from a drop in milk production in dairy cattle [99, 100, 179].

Sheep flocks are likely to be more susceptible to the impact of SBV infection than cattle herds due to the compact nature of the sheep breeding season [9]. These species-specific patterns are often influenced by the time interval in pregnancy during which transplacental infection can lead to foetal damage [180]. For example, early lambing sheep flocks are at greater risk of congenital Schmallenberg disease [181]; this is because the gestation susceptible period of early lambing ewes can overlap with the *Culicoides* vector-active season, enabling congenital SBV infection. This is consistent with reports of substantial losses associated with mortality and congenital malformations in early lambing sheep flocks [9, 95, 182]. In the Netherlands, deformities in lambs varied from mild to severe, and ewes were reported to have given birth to normal lambs, deformed lambs and dummy lambs that were unable to suckle; dystocia was also common [6]. In the UK, a sheep farmer survey that was conducted following the initial SBV epidemic reported that lamb and ewe losses were high on farms where SBV was confirmed or suspected [183]. In the same survey, the median number of lambs born (and the median number of deformed lambs) that died within one week of birth per 100 ewes were 18.2 (5.5) on farms where SBV was confirmed,11.3 (2.9) where SBV was suspected and 8.6 (0.0) where SBV was not suspected, respectively, while 8 to 16 per cent of SBV confirmed or suspected farms reported lamb mortality of ≥40 per cent [183]. Stokes et al. (2018) conducted a similar survey in the UK to determine the farm-level impact of SBV during the 2016–2017 lambing season and describe comparable results to the findings reported for the 2011/2012 outbreak; higher neonatal lamb mortality, dystocia and associated ewe deaths, and higher perceived impacts on sheep welfare, flock financial performance and farmer emotional wellness were reported on SBV confirmed (*n*=59) and SBV suspected (*n*=82), than SBV not suspected (*n*=74) farms [184].

In France the SBV impact in infected ruminant herds was primarily due to the birth of stillborns or deformed foetuses and neonates while the median frequency of morbidity was significantly higher in SBV-infected lambs compared to calves and kids; on average 8% of lambs, 3% of calves and 2% of kids were born with typical SBV malformations [97]. In the same French study, farmers reported retrospectively a lower prolificacy during the vector season, suggesting a potential impact of acute SBV infection during mating and early stages of gestation [97]. In Irish sheep flocks, the weaning rate in confirmed SBV-infected sheep flocks was found to be approximately 10% lower than in uninfected flocks [185]. Similarly, the results of a survey investigating the impact of SBV infection in sheep flocks in Belgium revealed a two-fold reduction in expected flock prolificacy in 2012 [105]. Preliminary studies on SBV infection in sheep flocks in France in 2012 reported mortality rates of up to 15%, albeit with considerable between-flock variation [186]. Congenitally malformed calves and lambs that survive are typically unsuitable for breeding or for sale, which has had an economic impact on farmers. The emotional impact of SBV infections has been also been reported; many sheep farmers with flocks affected by SBV reported distress due to the sight of congenitally malformed lambs and also stress associated with the emergence of a novel disease in their flocks [183]. While the overall economic impact of the SBV epidemic in Europe appears to be limited, the consequences have been substantial in farms with a high prevalence of clinical disease [106].

The most significant impact of Schmallenberg virus has been international trade restrictions, particularly in live animals and semen; a number of third countries (Non-EU) restricted cattle semen trade which resulted in an estimated drop of 11-26 % in the number of semen doses exported by Europe in 2012 [26]. Moreover, the official statistics (EUROSTAT) on semen exports from pure-bred breeding animals show that the export value dropped from almost 590 million Euros in 2011 (heifers, cows and other breeding animals) to 475 million Euros in 2012 (a decline of 20 per cent) [26].

The financial impact of SBV infection on individual livestock farms varies widely. One study in Belgium attempted to quantify the financial cost of SBV infections; the mean cost for individual symptomatic treatment of SBV-infected animals was estimated to be 65 and 107 Euros in the case of fatal outcome or apparent recovery, respectively [107]. Economic models have also been developed to estimate the financial costs of SBV infection on livestock farms in the UK and France; Häsler et al. (2015) developed a model to estimate the cost of SBV infection per cow space per year for an average dairy farm with 100 milking spaces in both high and low impact scenarios in the UK and in France [187]. The results showed that the net SBV disease costs in the UK in £/cow space/year was estimated to be £16.3 and £51.4 in the high impact scenario and between £8.2 and £25.9 in the low impact scenario, respectively [187]. In France, the net SBV disease costs in £/cow space/year ranged from £19.6 to £48.6 in the high impact scenario and £9.7 and £22.8 in the low impact scenario, respectively [187]. A similar model was developed for sheep flocks in the UK and in France [188]; the estimated net SBV disease cost per year and ewe was £19.65-£20.85 for the high impact scenario and £6.40-6.58 for the low impact scenario. No major differences were observed between the different production types [188]. For France, the net SBV disease cost per year and ewe for the meat and milk sheep holdings ranged from £15.59-£29.81 for the high impact scenario and £4.75-£10.34 for the low impact scenario depending on production type [188].

### Diagnostics

#### Virus detection

In congenitally malformed calves and lambs the preferred sample materials for detecting SBV RNA via RT-qPCR are brain stem, placenta, and meconium [167, 189]. Pre-colostral serum and foetal fluids can also be used to detect SBV-specific neutralising antibodies independently, or as an adjunct to tissue samples [167, 190]. For semen samples, frozen diluted or undiluted bull semen samples can be tested using RT-qPCR to detect SBV RNA [158–162]. Real-time reverse transcription quantitative polymerase chain reaction (RT-qPCR) is the method predominantly used for the direct detection of SBV antigen; due to the characteristics of the analysis such as high sensitivity, relatively time-efficient assay to perform and the possibility for high throughput screening. Different PCR systems have been developed to target either the S, M or L segment of the virus [3, 189, 191]; the S segment-based assay is considered the most suitable in terms of sensitivity and specificity for the detection of SBV RNA. A pan-Simbu RT-qPCR assay for the detection of a number of Simbu serogroup viruses has also been developed [191]. RT-qPCR assays are also used for the detection of SBV antigen in insect vectors [20, 137, 144–146, 192].

Virus isolation in various cell lines has also been used; SBV can be isolated on insect cell lines such as KC (*Culicoides variipennis* cell line), or mammalian cell lines such as Vero (African Green Monkey) or BHK (Baby Hamster Kidney) cells [3, 115].

#### Antibody detection

The detection of SBV-specific antibodies may be a more reliable diagnostic test in adult animals compared to virus detection considering the short duration of viraemia (approximately 4-6 days) and the non-specific clinical signs associated with SBV infection in adult animals [3, 92, 115, 117]. Moreover, the detection of SBV-specific antibodies in foetal heart blood (aborted foetuses) or in serum collected prior to ingestion of colostrum (neonates) can confirm congenital SBV infection [69, 167, 190]. Serum is the matrix of choice for detecting SBV RNA and antibodies in adult animals [26]. Milk (lacto-serum) samples can also be used to detect SBV-specific antibodies [193]. Similar to a number of other livestock diseases such as Bovine Viral Diarrhoea and Bovine Leukemia [194, 195], a number of research studies have investigated the relationship between SBV antibody titres in serum and milk samples. Bulk tank milk antibody titres are highly predictive of within-herd SBV seroprevalence and can be used as a surveillance tool to monitor SBV infection dynamics in dairy cattle herds [196]. One study demonstrated that antibody titres in individual animal milk samples were significantly higher when compared to serum samples in dairy cattle [197]. A number of countries in Europe have used BTM-ELISA results to monitor SBV infection dynamics in dairy herds [13, 198, 199]. Virus-specific antibodies can be detected in serum and milk using a variety of assays including in-house and commercial ELISAs (there are several commercial antibody ELISA-Kits available), micro-neutralisation and indirect immunofluorescence tests [193, 200–202]. A European-wide ring trial demonstrated limited inter-laboratory variation in the detection of SBV-specific antibodies in serum and that the virus neutralisation test was more sensitive compared to a number of different ELISAs [203]. While the SBV virus neutralisation test is highly specific [61, 204], the S-segment based ELISAs could lead to cross-reactivity between SBV and other Simbu viruses [114]. This is particularly important to consider in the context of selecting the most appropriate diagnostic tests to use in order to confirm the emergence of SBV in new geographical regions. Currently (September 2019), SBV is the only known Simbu serogroup Orthobunyavirus virus reported in Europe.

### Surveillance

A number of surveillance systems were established across Europe in order to monitor for SBV (re-)emergence and (re-)circulation. These included syndromic surveillance, sentinel herd surveillance, cross-sectional seroprevalence studies and pathogen surveillance in animals and vectors. In the Netherlands a syndromic surveillance system based on routinely collected cattle reproduction and milk production data proved effective for the early detection of outbreaks of Bluetongue and Schmallenberg viruses [205]. Regional sentinel veterinarians were also used in a France to monitor suspect SBV cases (arthrogryposis-hydranencephaly syndrome) in ruminants [206]. A *Culicoides* dispersion model has been developed by DAFM in conjunction with the Irish Meteorological Office (Met Éireann) and UCD Centre for Veterinary Epidemiology and Risk Analysis to monitor weather conditions which may favour a possible incursion of *Culicoides* from the UK and continental Europe [68]. However, it is recognised that microclimatic temperatures provide better estimates of vector-borne disease transmission parameters when compared to standard meteorological temperatures, as the microclimate represents the actual temperatures to which the vectors are exposed [136]. Vector-borne disease transmission models for Schmallenbreg virus commonly use mathematical equations originally developed for Bluetongue virus serotype 9, for example Bessel et al. (2014) [136, 181] and Haider et al. (2018). This is often because there are no specific experimental data on the relationship between temperature and virus replication rate (extrinsic incubation period) in *Culicoides* midges for SBV. Further research in this area is recommended in order to determine the microclimatic conditions which are favourable for SBV replication in *Culicoides* species; this could enable more reliable predictions of SBV epidemics.

Within the first 2.5 years of the emergence of the first SBV cases in Germany in 2011, the virus had achieved an almost pan-European distribution (Fig. 1). To date (September 2019), the virus has been confirmed in almost all European countries including Austria [98], Belgium [108], Croatia [207], Denmark [143], France [165], Germany [3], Great Britain (England, Scotland and Wales) [27, 208], Greece [209], Hungary [50], Ireland [11], Italy [210], Luxembourg, Latvia, Estonia, Finland and the Czech Republic [211], The Netherlands [6], Northern Ireland [212], Norway [213], Portugal [214], Romania [215], Serbia [216], Slovenia [217], Spain [218], Sweden [89, 199], Switzerland [19] and Turkey [10]. It is likely that the neighbouring countries have also had SBV infections but confirmed cases have not yet been reported. A model for the transmission of SBV between regions in Europe suggested that vector dispersal is the principal mechanism for transmission of SBV, even at the continental scale [219]. More recently, SBV has been reported in countries outside of Europe, including Azerbaijan [31], China [32], Ethiopia [33, 34], Iran [35], Lebanon [36], Namibia [38] and Mozambique [31, 37] suggesting possible geographical expansion of SBV. However, reports of SBV emergence in new geographical regions which are based solely on serological detection of SBV antibodies, particularly when samples originate in regions where other Simbu viruses are known to be enzootic, should be interpreted cautiously as some ELISAs can have cross-reactivity between SBV and other Simbu viruses [114].

Schmallenberg virus re-emergence and recirculation occurred in cattle herds in Ireland [106] and in the UK [27, 184, 220] during 2016 and 2017 resulting in a drop in milk yield and congenital malformations in calves and lambs. More recently (January/February 2018), SBV infection was also confirmed in malformed bovine foetuses in Ireland [106]. While a number of these cases are in livestock in regions where SBV had circulated previously, confirmed reports of SBV emergence in the north and north west of Ireland (where the virus had not been detected previously), suggests geographical expansion of SBV into regions with little, if any, SBV herd or flock immunity [30]. Reports of SBV re-emergence and recirculation during 2017 is restricted to Ireland and the UK; this may be due to lack of notable virus circulation (possibly due to high herd immunity/endemic circulation) in continental Europe or possibly underreporting/cases not yet reported. Further research is recommended to determine the current herd immunity to SBV in Europe; this information can in turn be used by policy makers, veterinarians and farmers to inform decisions regarding the future risk of SBV circulation and possible epidemics.

A sentinel surveillance program to monitor SBV infection in Irish cattle herds proved very effective in monitoring for SBV recirculation and re-emergence [20, 28]. This type of surveillance model is not unique, in fact a number of countries have established similar arbovirus monitoring/surveillance programs, typically at a national level. For example, in Australia, the National Arbovirus Monitoring Program (NAMP) is very effective in monitoring arboviruses such as Akabane, bluetongue and bovine ephemeral fever viruses [221]. The NAMP model monitors sentinel farms throughout the country (in endemic areas, border regions and disease/vector-free areas) on a permanent basis using serology, virus isolation and vector surveillance (*Culicoides* species) [221]. Future arbovirus surveillance work in Ireland and in Europe could be based on such a program; the establishment of a European-wide sentinel herd surveillance program, which incorporates bovine serology and *Culicoides* entomology and virology studies to monitor for the emergence and re-emergence of arboviruses such as SBV, bluetongue virus and other novel *Culicoides*-borne arboviruses is recommended.

### Control and prophylaxis

In response to the European Schmallenberg epidemic in 2011-2012, a number of research studies aimed to develop suitable SBV vaccines for use in domestic ruminants to protect against SBV infection. Wernike et al. (2013) developed four vaccine prototypes which completely prevented viraemia in cows after challenge infection [222] while Hechinger et al. (2014) developed and inactivated SBV vaccine requiring only a single immunization in sheep resulting in complete inhibition of viral replication in immunized animals [223]. A double deletion mutant of Schmallenberg virus (modified live vaccine) was also reported avirulent and protected animals against SBV infection [224]. Subsequently three inactivated commercial vaccines (Zoetis Zulvac [Zoetis Belgium SA, Rue Laid Burniat 1, 1348 Louvain-la-Neuve, Belgium], Bovilis® SBV [Intervet UK Ltd. Milton Keynes, Buchinghamshire, United Kingdom] and SBVvax [Merial SAS, Lyon, France]) were developed for use in cattle and sheep. These vaccines were marketed in France and the UK in 2013, in the Republic of Ireland in 2014, and later marketed to the rest of the Europe in May 2015. In Scotland, one study which developed a stochastic mathematical model of SBV spread to investigate the optimal deployment of a vaccine found that SBV vaccine impact is optimised by targeting it at high risk areas or vaccinating only cattle [181]. The same study also demonstrated that at higher than average temperatures, and hence increased *Culicoides* transmission potential, the relative impact of vaccination was also considerably enhanced [181]. Despite the initial uptake of vaccine among veterinarians and farmers in Ireland, both vaccines were subsequently withdrawn from the Irish market due to a reduction in demand. Currently (September 2019) there is no SBV vaccine licenced for use in Ireland. There is no specific treatment available for SBV infection.

Alternative, but less reliable control measures have been proposed. As SBV is an insect-transmitted virus, the use of insecticides or repellents directed against vectors could, in theory, be useful to prevent virus transmission from virus-infected midges to susceptible animals. However, one case-control study demonstrated no evidence of protection from such treatments against SBV infection [170]. Strategic management of the breeding season has also been suggested [95]; adjusting the breeding season to avoid having animals at the most critical phase of gestation during the period when SBV arbovirus vectors are most active (vector-active period spans from April to November in Ireland), may help reduce the possibility of virus transmission. However, this could result in significant implications for both sheep and cattle management in seasonal-based production systems (such as in Ireland). Moreover, changing breeding times for livestock is likely to have economic implications for farmers, particularly for early lambing sheep farms.

In addition to this, grazing/managing youngstock outside during the vector-active season may help facilitate exposure to vectors, possibly resulting in natural SBV infection. In this instance, young animals are more likely to be exposed to SBV before their first breeding season. Considering that naturally acquired SBV immunity is thought to be long-lasting; anti-SBV antibodies are detectable for at least 2-3 years following natural infection cattle and sheep [116–118] the immunity acquired as a calf/lamb may help prevent SBV infection in breeding/pregnant females later in life. However, bearing in mind the inconsistent and intermittent circulation of SBV in previously exposed regions following the European Schmallenberg epidemic in 2011/2012, this method is unlikely be very reliable on its own. Rather, a combination of a number of control measures is required to reduce the risk of SBV infection in domestic livestock.

## Conclusions

Since SBV was first identified in 2011, a considerable body of scientific research has been conducted internationally on this novel emerging virus. This review provides a comprehensive synopsis of the most up-to-date scientific literature regarding SBV internationally. Moreover, the review also highlights current knowledge gaps in the literature, most notably the need for further research to determine if, and to what extent, SBV circulation occurred in Europe during 2017 and 2018. This information will be critical to determine the current herd immunity to SBV in Europe and can in turn be used by policy makers, veterinarians and farmers to inform decisions regarding the future risk of SBV circulation and epidemics. The authors of this review recommend that SBV circulation in continental European countries during 2017 and 2018 be investigated.

The results of this review also highlight that currently, there is no commercial vaccine available to protect domestic ruminants against SBV infection, and historically when a vaccine was available, uptake was low. It would therefore be prudent to continue monitoring for Schmallenberg virus circulation in previously exposed and unexposed regions in Europe. Moreover, the establishment of a European-wide sentinel herd arbovirus surveillance program, which incorporates bovine serology and *Culicoides* entomology and virology studies, at national and international level to monitor for the emergence and re-emergence of arboviruses such as SBV, bluetongue virus or other novel *Culicoides*-borne arboviruses, is also recommended.

## Data Availability

Not applicable.

## Abbreviations

AHS: Arthrogryposis Hydranencephaly Syndrome
AIV: Aino virus
AKAV: Akabane virus
BHK: Baby Hamster Kidney
BTM: Bulk Tank Milk
BTV: Bluetongue virus
BVDv: Bovine Viral Diarrhoea virus
CNS: Central Nervous System
Ct: Cycle threshold
DAFM: Department of Agriculture, Food and the Marine
ECE: Embryonated Chicken Egg
EIP: Extrinsic Incubation Period
ELISA: Enzyme Linked Immunosorbant Assay
Gc: Glygoprotein C
Gn: Glygoprotein N
IFN: Interferon
IFNAR-/-: Type 1 Interferon Knockout Mice
IIP: Intrinsic Incubation Period
L: Large
M: Medium
NSm: Nonstructural protein
RT-qPCR: Reverse Transcriptase quantative Polymerase Chain Reaction
S: Small
SBV: Schmallenberg virus
SHAV: Shamonda virus
UK: United Kingdom
